# Using climate envelopes and earth system model simulations for assessing climate change induced forest vulnerability

**DOI:** 10.1038/s41598-024-68181-5

**Published:** 2024-07-24

**Authors:** Leam Martes, Peter Pfleiderer, Michael Köhl, Jana Sillmann

**Affiliations:** 1 Institute for Wood Science - World Forestry, Universität Hamburg, Leuschnerstraße 91, 21029 Hamburg, Germany; 2 Research Unit for Sustainability and Climate Risks, Universität Hamburg, Grindelberg 5, 20144 Hamburg, Germany; 3 Climate Analytics, Berlin, Germany

**Keywords:** Forest, Climate envelopes, Climate change, Climate extremes, Tree mortality, Vulnerability, Climate-change ecology, Forest ecology, Forestry, Climate sciences

## Abstract

Changing climatic conditions threaten forest ecosystems. Drought, disease and infestation, are leading to forest die-offs which cause substantial economic and ecological losses. In central Europe, this is especially relevant for commercially important coniferous tree species. This study uses climate envelope exceedance (CEE) to approximate species risk under different future climate scenarios. To achieve this, we used current species presence-absence and historical climate data, coupled with future climate scenarios from various Earth System Models. Climate scenarios tended towards drier and warmer conditions, causing strong CEEs especially for spruce. However, we show that annual averages of temperature and precipitation obscure climate extremes. Including climate extremes reveals a broader increase in CEEs across all tree species. Our study shows that the consideration of climate extremes, which cannot be adequately reflected in annual averages, leads to a different assessment of the risk of forests and thus the options for adapting to climate change.

## Introduction

Forests occupy one third of the Earths land area, which shows that they can persist under a wide amplitude of climatic conditions. As long as the climate remains stable over a longer period of time, forest ecosystems have the ability to adjust to climatic conditions and other environmental factors such as competition or detrimental biotic and abiotic impacts^1^. Historically, forests have repeatedly adapted to changing climatic conditions^2^. Under current climate change, shifts in the spatial distribution of tree species, interspecific competition, and ultimately forest composition are expected^3–6^. This will be accompanied by changes in the provision of ecosystem goods and services^7,8^.

Due to their sedentary nature and long lifetime relative to other organisms^9–11^ the rate at which current climate change is occurring presents a particular challenge to the adaptive capacity of trees^12^. A natural response to climate change through habitat displacement by tree migration is proving critical due to the described post-glacial migration rates of 60–260 m y$^{-1}$^13^.

Climate change is already having an impact in the current range of forests. In addition to ongoing changes in temperature and precipitation patterns, climate extremes have a particular impact^14^. Higher temperatures and increasing droughts will lead to an increase in natural disturbances that affect forest vitality and health. These events include not only heat waves, droughts and the associated forest fires^15–18^, but also disease, and insect plagues^19–24^, which are projected to increase in frequency and intensity as well^25^.

In Central Europe a substantial amount of drought-weakened growing stock was destroyed by windstorms and spruce bark beetles during the dry years from 2018 to 2020^26,27^. While the damage is spread across many countries, the greatest losses are found in Germany, Czechia, and Austria. In Germany, for the years 2018 to 2022, a calamity wood accumulation of 255 million m$^{3}$ has been recorded, representing 7% of the 2015 annual increment, or more than 3 times average annual harvest^28^, of which 233 million m$^{3}$ are coniferous and 22 million m$^{3}$ are deciduous. In Germany alone, the forest area to be reforested is over 490,000 hectares^29,30^, which amounts to approximately 5% of total forest area^28^. Global climate change is likely leading to more frequent disruptions in other European regions as well^31,32^, with drought becoming particularly prominent^16,33,34^.

The frequency of mortality tends to increase when a species is outside of its biological optimum. This optimum is a combination of a large number of environmental factors, including temperature, rainfall, altitude and substrate, which together form an area where a species can thrive. Ellenberg used these relationships to derive index values for individual plant species that evaluate the real occurrence of the species in the field^35^. The approach of index values was further developed to so-called climate envelope models which according to Watling et al.^36^ refer to: ”a subset of species distribution models that use climate variables to make spatial predictions of environmental suitability for a species”. The climate envelope approach has been criticized, as it relates to the climate-space only and does not reflect the interaction of species which can be altered by climate^37,38^ . Nevertheless climatic envelopes have been applied to facilitate the understanding of current and future dispersal of species and to identify areas where species occur today but where their climatic requirements will no longer be met in the future^6,39^.

Most forests in Europe are managed semi-natural forest areas. Current managers face a difficult choice when restocking forest stands, regarding which species they can rely upon in the future. Currently there is a reliance on natural regeneration of existing tree species. It is therefore important to identify the most vulnerable tree species, and to quantify the risks forest managers must deal with in regards to future forest for vitality and health under changing climate conditions. Some coniferous tree species, such as *Picea abies* (Norway spruce) and *Pinus sylvestris* (Scots pine) were historically planted because of their relatively fast growth and high timber quality in the local climate^40^, but now might be threatened due to changing climatic conditions^41,42^. *P. abies* especially has faced high mortality in the past decade due to ongoing drought coupled with insect plagues^43–45^. This species is of particular importance, due to its relatively high area coverage (around 26% in Germany) coupled with its high commercial value^46^.

While previous studies have looked at the general trends in tree species distribution under different climate scenarios^47–49^ this study will evaluate the frequency of years with climatic conditions outside of the species-specific climate envelopes based on historical climate data and state of the art climate projections. We use Climate Envelope Exceedance (CEE) frequency to assess relative vulnerability to climatic change between four commercially important tree species. Using this approach the vulnerability of tree species to changing climate conditions can be assessed more comprehensively by also considering the effect of extreme weather events. We use Europe-wide historical climate data to calculate the species-specific climate envelopes and combine them with climate predictions from Earth System Models (ESMs) for a case-study site in the Hamburg metropolitan area. We limit the range of the future climate scenarios to one specific case study site in order to gather the frequency of climatic changes without needing to summarize future climate data either spatially or temporally.

An increase in CEEs over time means that the climate is drifting away from a species optimum range. As climate variables surpass the bounds of the tree species’ climate envelopes, trees become more vulnerable to the previously mentioned climate stressors^50^. The inclusion of multiple bio-climatic variables will aid in more precisely identifying which changing climatic factors could drive increased stress, and by extension mortality, in the future under different climate scenarios, and allow us to identify which species are more vulnerable than others to changing climate conditions. The results can be used to guide management practices to take measures to mitigation future risks related to forest degradation and mortality, as well as the selection of suitable adaptation strategies such as assisted adaptation.

## Methods

### Tree occurrence data

Tree occurrence data was obtained from the EU-Forest data set by Mauri et al.^51^, which is a harmonisation of forest plot surveys from various national forest inventories from the EU, EFTA, and the United Kingdom, organised in an INSPIRE compliant 1$\times$1 km grid. The following four tree species were used from the data set:

- Norway spruce (*Picea abies*)
- Scots pine (*Pinus sylvestris*)
- European beech (*Fagus sylvatica*)
- Pedunculate oak (*Quercus robur*)

**Figure 1:**
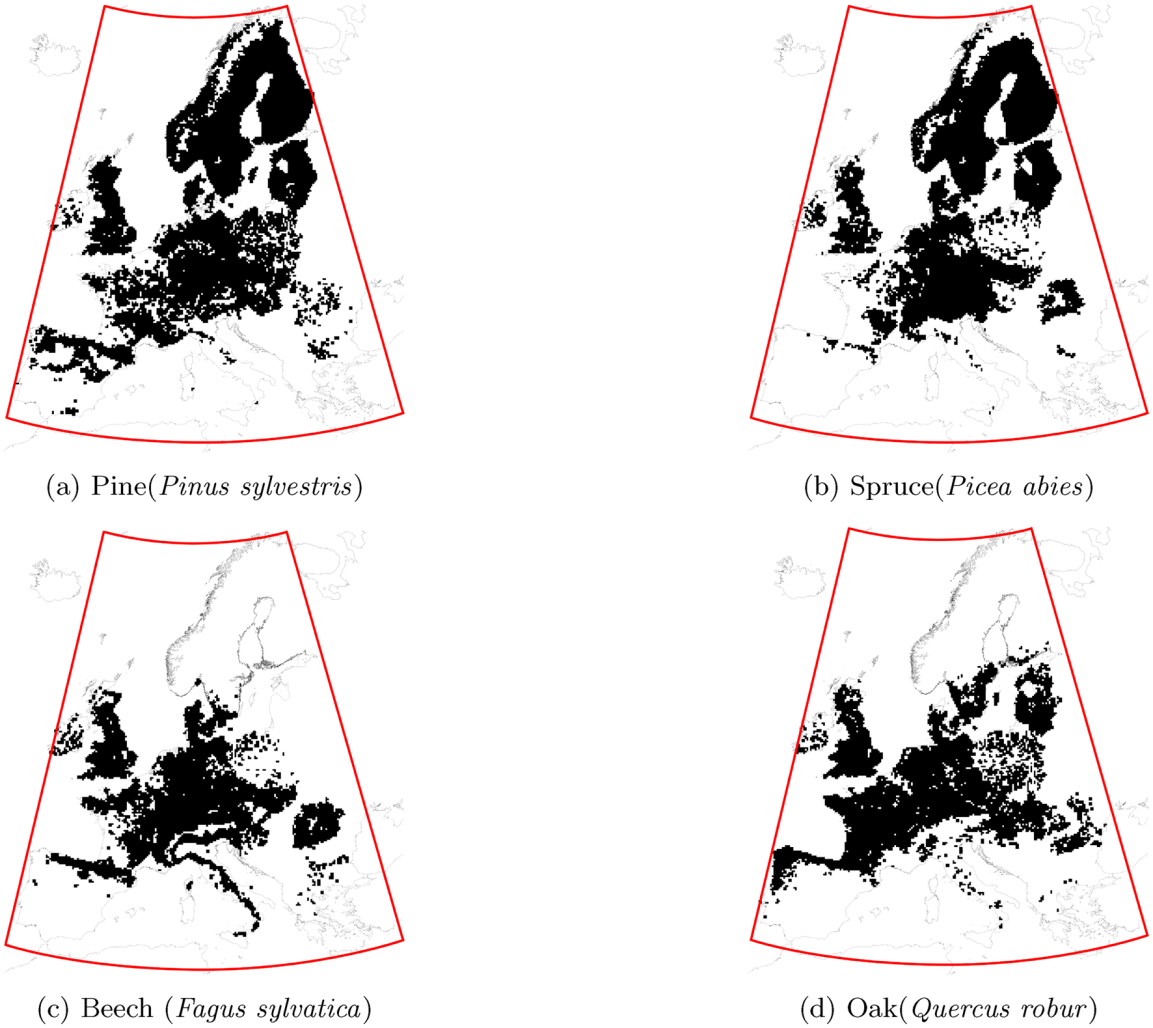
Presence-absence data for the 4 selected tree species, with the relevant study area for the historical climate data outlined in red.

We then transformed the species occurrence data into a presence absence raster with a resolution of 0.25 by 0.25$^{\circ }$ (see Fig. 1) in order to unify the different sampling densities of the individual national forest inventories that comprise the dataset by Mauri et al.^51^.

### Climate data

We collected climate data from the ERA5 reanalysis data set: “hourly data on single levels from 1940 to present” by Herschbach et al.^52^. The variables used were 2-metre temperature (t2m) and total precipitation. Data was gathered for the time period of 01-01-1960 until 31-12-2020. The area covered the whole European continent and is delineated by the following coordinates: North: 71.1$^\circ$, West: − 9.5$^\circ$, South: 34.5$^\circ$ and East: 31$^\circ$ with a resolution of 0.25 by 0.25$^\circ$, outlined above the presence-absence data in Fig. 1. The climate data was then extrapolated over the entire grid area using an Inverse Distance Weighted (IDW) interpolation. We then compiled the hourly temperature and precipitation values in to four monthly variables: minimum monthly temperature, maximum monthly temperature, average monthly temperature and total monthly precipitation. Using these aggregated variables, we calculated 19 bioclimatic variables according to the methods published by the US Geological Survey^53^. These 19 bioclimatic variables can be found in Table 1. We then interpolated these 19 bioclimatic variables in to an 0.25 by 0.25 degree resolution raster using an inverse distance interpolation.

**Table 1:** The 19 Bioclimatic variables used in this study, adapted from^47^.

Parameter	Description
BIO1	Annual mean temperature [°C]
BIO2	Mean diurnal range [°C]
BIO3	Isothermality (BIO2/BIO7)$\cdot$100
BIO4	Temperature seasonality (sd$\cdot$100) [°C]
BIO5	Max temperature of the warmest month [°C]
BIO6	Min temperature of the coldest month [°C]
BIO7	Temperature annual range (BIO5–BIO6) [°C]
BIO8	Mean temperature of the wettest quarter [°C]
BIO9	Mean temperature of the driest quarter [°C]
BIO10	Mean Temperature of the Warmest Quarter [°C]
BIO11	Mean temperature of the coldest quarter [°C]
BIO12	Annual precipitation [mm]
BIO13	Precipitation of the wettest month [mm]
BIO14	Precipitation of the driest month [mm]
BIO15	Precipitation seasonality (mean_y_/sd_m_) $\cdot$100 [mm]
BIO16	Precipitation of wettest quarter [mm]
BIO17	Precipitation of driest quarter [mm]
BIO18	Precipitation of warmest quarter [mm]
BIO19	Precipitation of coldest quarter [mm]

By overlaying two different data sets and masking to exclude sea surface, it was possible to attain the climatic variables associated with each instance of the presence absence raster with the 19 bioclimatic variables.

### Statistical analysis

Next, we conducted a Non-metric Multidimensional Scaling (NMDS)^54^ analysis to create a dissimilarity matrix of the four tree species and 19 bioclimatic variables. This method was chosen as it performs better with non linear data such as the ecological presence-absence data obtained in this study^55^. An NMDS model is an ordination metric that tries to represent dissimilarity between groups, in our case the four selected tree species, using the 19 Bioclimatic variables, grouped in to so called dimensions as the separating variables. We use this analysis to determine if the four tree species are significantly different regarding their occurrence in relation to the 19 bioclimatic variables, and which of the bioclimatic variables are more associated with the two available axes of difference. We then make a selection of the relevant bioclimatic variables to continue with in the analysis.

These selected bioclimatic variables are then used in a Species Distribution Model (SDM) were each species occurrence is predicted over each response variable , assuming a unimodal distribution of said response variable. To obtain the range of each species over the response variables, we use a cutoff occurrence level of 0.1.

### Future climate data

We assess possible future changes in bioclimatic variables in Earth system model (ESM) simulations of the 6^th^ phase of the Coupled Model Intercomparison Project (CMIP6)^56^ for the area around Hamburg in Northern Germany. This was chosen as a case study representative of the Northern European temperate zone. The CMIP6 models we used were a sub-selection based an on impact assessment for Europe conducted by Palmer et al.^57^. Instead of analysing climatic changes for specific emission scenarios, we focus on global warming levels (GWL) relative to pre-industrial levels. These GWLs are based annually averaged global mean surface air temperature (GMST) relative to a pre-industrial period 1850–1900. Using GWLs allow us to pool climate projections from different scenarios therefore increasing the robustness of the results, and also allows us to compare results irrespective of their underlying emission scenarios. For each of the GWLs in 1.2 $^\circ$C (ref), 1.5 $^\circ$C, 2 $^\circ$C, 2.5 $^\circ$C and 3 $^\circ$C and each ESM we aggregate 30-year periods that match the GWL from different simulations that have been produced by the ESM. Thereby we obtain longer periods (length depends on the number of available simulation runs) with similar climatic conditions representing a certain GWL.

ESM are calibrated to represent climatic conditions around the globe and therefore biases on the local level are inevitable. To allow comparability with the ERA5 reanalysis we perform a quantile mapping bias adjustment^58^. This method adjusts the distribution of a climate variable to a reference dataset over a reference period (1980–2010) without influencing the trend in the variable simulated by the ESM.

### Climate envelopes

We calculated CEEs for all climate scenarios per tree species. Exceedance years are years in which the predicted yearly average of a bioclimatic has exceeded the climate envelope of a tree species. Exceedance years can be positive (above the maximum envelope value) or negative (below the minimum envelope value). We then categorized each exceedance year according to its intensity level, which is the percentage of deviation from the climate envelope, in relation to the size of the envelope. These exceedance range from > 0–5, 5–10, 10–15, 15–25, 25–30 and > 30% of envelope size exceeded. Finally we summarized all exceedances into a percentage based risk factor, this reflects the percentage of years in which the envelope has been exceeded, for each of the five GWLs.

## Results

**Figure 2:**
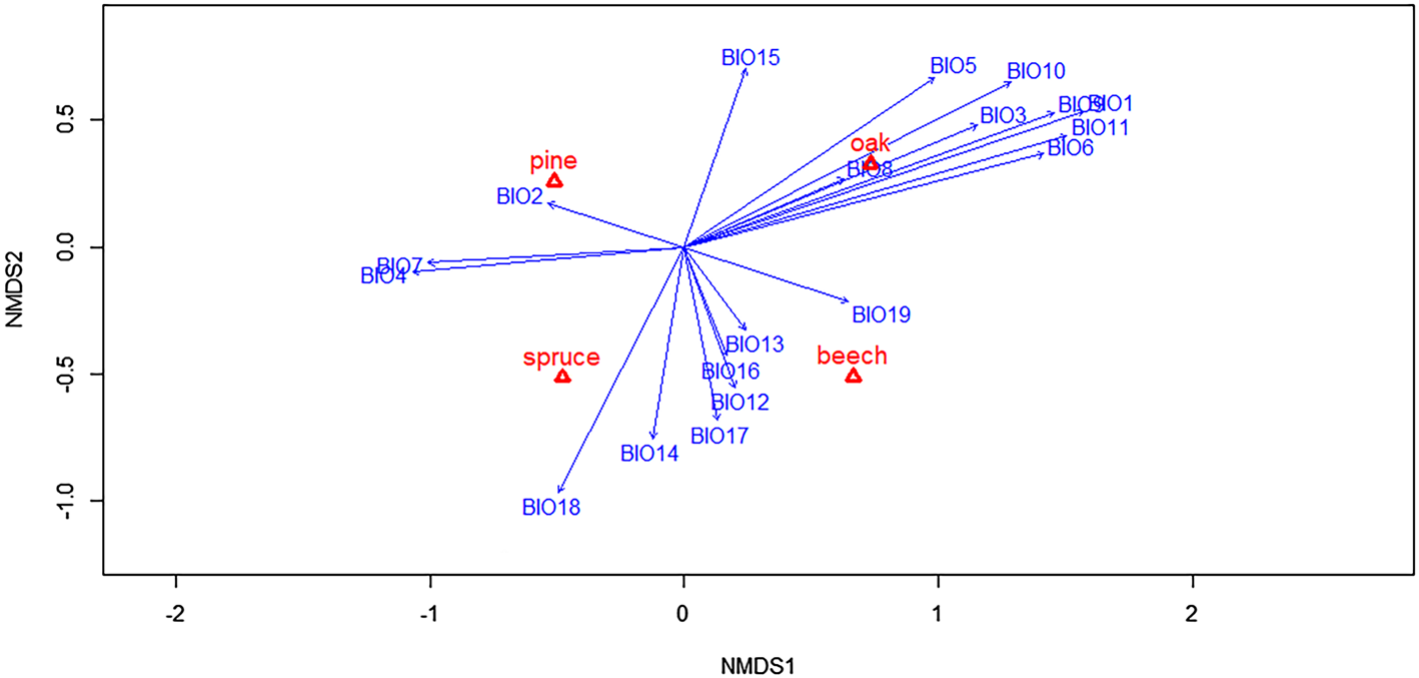
The graphical representation of the Non metric Multi-Dimensional scaling (NMDS) analysis performed on the 4 tree species and for all 19 bioclimatic variables.

The Non-Metric Multidimensional Scaling (NMDS) analysis, was run with a dimensionality of 2, using the Jaccard Similarity method. The analysis resulted in a stress of 0.064074. The results of the model are shown in Fig. 2, we found that all four included tree species (Norway spruce, Scots pine, Pedunculate oak and European beech) were sufficiently different from one another other on both the horizontal and vertical axis. The negative vertical axis most aligned with Precipitation in the warmest quarter (BIO 18), Precipitation in the driest month (BIO 14), Precipitation of the driest Quarter (Bio 17), Annual precipitation (BIO 12), Precipitation of the Wettest Quarter (Bio 16) and Precipitation of the Wettest Month (BIO 13). The vertical positive axis is slightly associated with Precipitation Seasonality (BIO 15). The horizontal negative axis was most correlated with Temperature Seasonality (BIO 4), Mean Diurnal Range (BIO 2) and Temperature Annual Range (BIO 7). The horizontal positive with Max. Temperature of the Warmest Month (BIO 5), Isothermality (BIO 3), Mean Temperature of the Wettest Quarter (BIO 8), Min. Temperature of the Coldest Month (BIO 6), Mean Temperature of the Driest Quarter (BIO 9), Annual Mean Temperature (BIO 1), Mean Temperature of the Warmest Quarter (BIO 10) and Mean Temperature of the Coldest Quarter (BIO 11). We could therefore derive that NMDS1 more strongly represents the temperature variables and NMDS2 the precipitation variables. For a full numerical overview of the NMDS analysis consult Table S1 in the supplementary information.

Of these 19 variables seven were selected for further analysis, BIO1 and BIO12 were included by default, as they represent the yearly averages of temperature and precipitation respectively and are used as a baseline of comparison. The other 5 variables were based on their correlation with the two major axes. These were, Temperature Seasonality (BIO4), Max. Temperature of the Warmest Month (BIO 5), Precipitation Seasonality (BIO 15), Precipitation of Warmest Quarter (BIO 18) and Precipitation of Coldest Quarter (BIO 19). These seven variables were used in the final SDM. The cutoff range for species occurrence was set at a probability of 0.1. All General Linear Models showed statistically significant coefficients and had a p-value < 0.001. The final ranges can be found in Table 2. For a graphical overview of al GLM results see the supplementary materials Figs. S2–S9.

**Table 2:** The upper and lower ranges as well as the average for each of the four tree species for all seven selected bioclimatic variables: BIO1 (Annual Mean Temperature), BIO4 (Temperature Seasonality), BIO5 (Max. Temperature of the Warmest Month), BIO12 (Annual Precipitation), BIO15 (Precipitation Seasonality), BIO18 (Precipitation of the Warmest Quarter) and BIO19 (Precipitation of the Coldest Quarter).

Species		BIO1 [°C]	BIO4 [°C]	BIO5 [°C]	BIO12 [mm]	BIO15 [mm]	BIO18 [mm]	BIO19 [mm]
Pine	upper	15.5	–*	35.3	2585.7	76.6	1042.3	–*
	lower	− 6.8	3.1	18.6	457.1	–*	103.8	–*
Spruce	upper	12.8	–*	31.1	2582.9	58.3	873.1	–*
	lower	− 6.9	3.0	17.3	578.6	–*	180.8	- -*
Beech	upper	13.8	8.0	35.1	1557.1	72.3	1321.2	388.5
	lower	6.7	2.1	23.9	781.3	–*	125	157.7
Oak	upper	12.2	9.0	36.15	1571.4	71.9	430.8	488.1
	lower	5.6	0.0**	23.6	657.1	–*	90.4	96.2

*These tree species did not show a unimodal distribution for their respective climatic variables, therefore 1 or even no envelope cutoff point was found. **Value was negative, but changed to 0 as seasonality can not be lower than 0.

**Figure 3:**
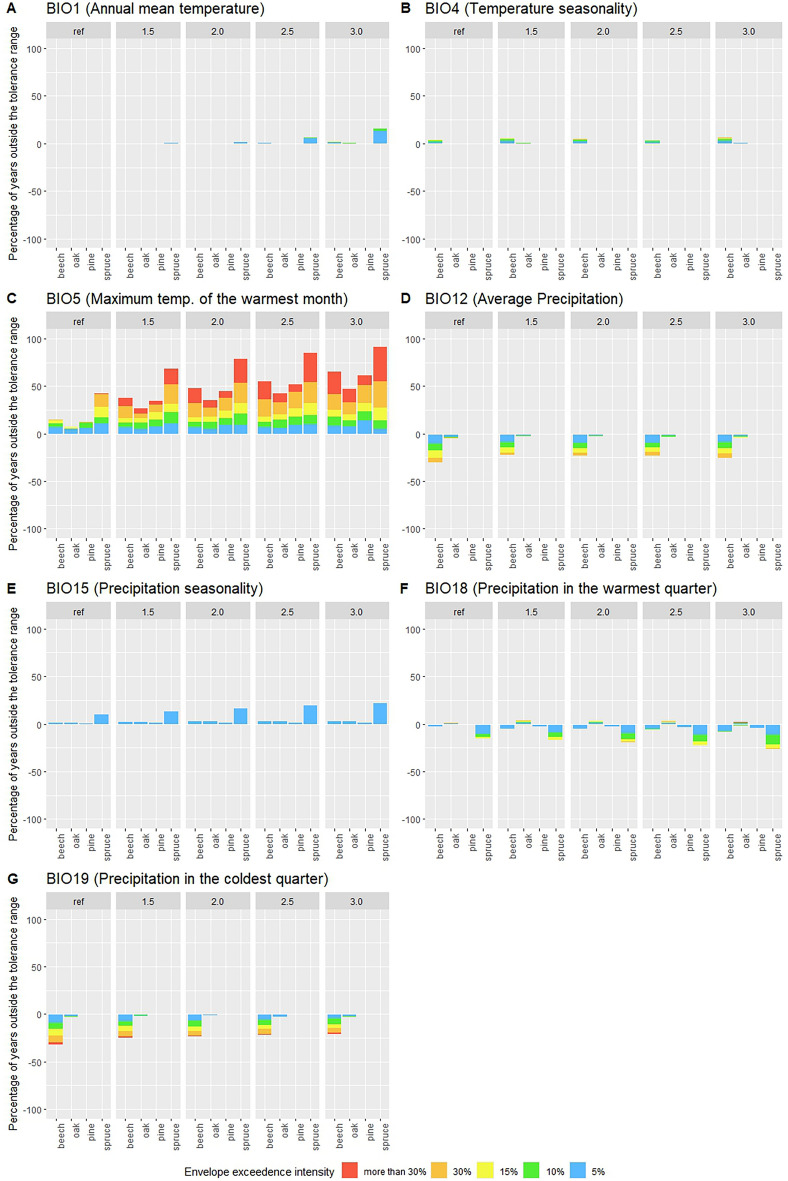
CEEs summarized as percentage based risk factors for the 4 tree species for all 7 selected bioclimatic variables. Divided over the reference period and the 4 GWLs. (**a**) (BIO1) Average yearly temperature. (**b**) (BIO4) Temperature seasonality. (**c**) (BIO5) Maximum temperature of the warmest month. (**d**) (BIO12) Average yearly precipitation. (**e**) (BIO15) Precipitation seasonality. (**f**) (BIO18) Average precipitation of the warmest quarter. (**g**) (BIO19) Average precipitation of the coldest Quarter. The five colours represent envelope drift exceedance of up to 5% (blue), between 5 and 10% (green), between 10 and 15% (yellow), between 15 and 30% (orange) and more than 30% (red). With the y axis being the frequency of envelope drift, divided in to positive and negative exceedance.

For the first climate envelope in the analysis (BIO1), as seen in Fig. 3a which represents the mean annual temperature we can see only positive Climate Envelope Exceedance (CEE). This means that some scenarios exceeded the upper range of annual mean temperature tolerance. In the reference scenarios (1980–2010) the mean temperature is never surpassed. This does however occur for spruce in a few years with a Global Warming Level (GWL) = 1.5 $^\circ$C, and around 10% of all years with a GWL = 2.5 $^\circ$C. At GWL 3.0 $^\circ$C beech and oak as well as spruce see some positive CEE of the upper bound of BIO1, with spruce continuing to be the most prominently affected with slightly under 25% of all occurrences of all years seeing envelope exceedance. Far lower are the CEE frequencies of pine followed by oak and beech, With beech CEE being slightly more frequent than oak.

The next variable BIO4, seen in Fig. 3b relates to the annual temperature seasonality (difference between the lowest and highest temperature of the year). Here we find little difference when comparing with reference, with only beech showing a slight increase in positive CEE.

When looking at the next variable BIO5 (Fig. 3c) which represents the maximum annual temperature, we see that there are already significant CEE in the reference scenario. Throughout increasing GWLs we see a large increase in CEE up to more than than 50% for all tree species. CEE, with more than a third all years exceeding the envelope by more than 30%.

For the variable BIO12, as seen in Fig. 3d that represents average annual precipitation, we can observe a slight trend in negative CEE, i.e. years with less that the lower bound of rainfall per year. Here beech is most affected although not significantly different from the reference.

For BIO15 (Precipitation Seasonality), seen in Fig. 3e, we can see a slight increase in CEE from increasing seasonality mainly for spruce.

For the variable BIO18, seen in Fig. 3f, which is the total precipitation in the warmest quarter of the year, we see that there is both slight positive, but mainly negative CEEs. Compared to the reference, negative CEEs show a slight increase with increasing GWLs primarily for spruce, while positive CEEs seen for oak remains relatively stable. Spruce is again the most prone to negative CEEs in all scenarios, increasing from a chance of 15 to 25 % throughout the five GWLs. Again, as with BIO5, the increase in frequency is associated with a growth in intensity as well.

Finally with the variable BIO19, as seen in Fig. 3g, associated with the precipitation in the coldest month. Here there is a slight trend in decreasing negative CEE seen for beech.

**Figure 4:**
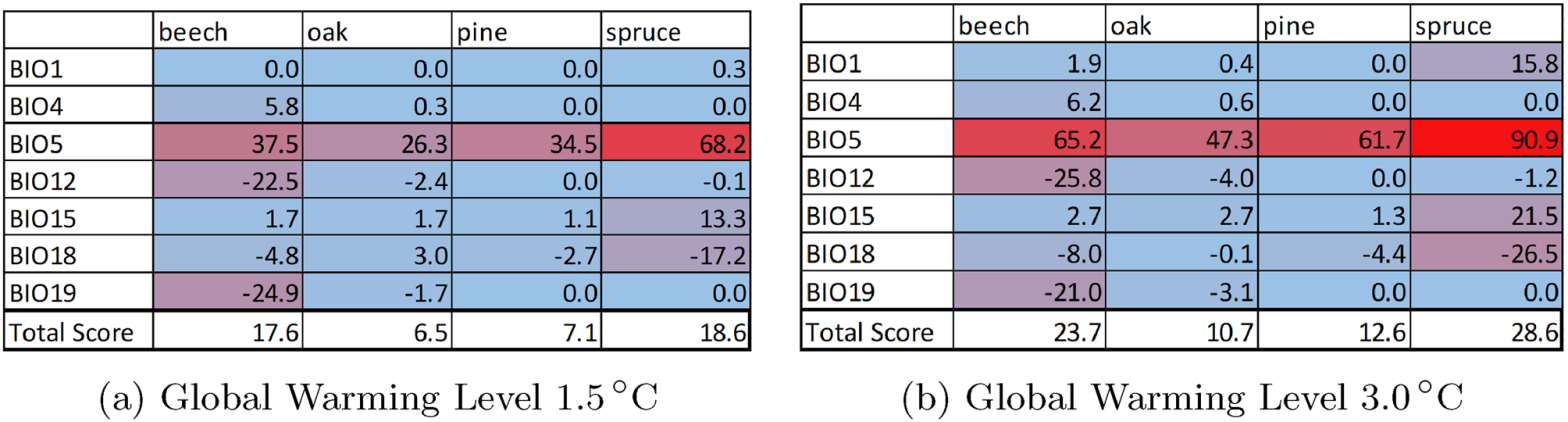
Averaged values of CEEs as percentage based risk factors for all tree species and bioclimatic variables, for the GWLs 1.5 and 3.0 $^\circ$C with their respective total scores.

In Fig. 4 we compile all the individual envelope exceedences into a percentage based risk factor. The total score was calculated as the sum of all absolute values weighed by the sum of their values on both ordination axes (see supplementary Table S1). Spruce is the tree species with the largest amount of CEE overall, for Global Warming Levels 1.5 $^\circ$C through 3.0 $^\circ$C. However, all species show large CEEs for BIO5. With Spruce approaching 90% of years exceeding BIO5 (Max. temperature of the warmest month), and the other tree having a CEE of between 47 and 65% for GWL 3.0 $^\circ$C. Total envelope exceedence scores all increase significantly for all four tree species between GWL 1.5 $^\circ$C and 3.0 $^\circ$C.

## Discussion

We assessed the vulnerability of four tree species to climatic change by using CEE frequency and intensity for an array of future climate scenarios. Similar studies looking at climate envelopes for European tree species, investigated how species occurrence will shift as climate change progresses^6,47^. The approach of our research was not to predict range shifts, but to use climate envelopes as a proxy for tree species vulnerability. We make the assumption that stress increases when climate envelope boundaries are exceeded. When we then compare the frequency of envelope exceedences between tree species and for each significant climate variable, we can then asses which species are more vulnerable than others to climatic changes, and which of said changes are most likely to cause an increase in vulnerability. This approach, using multiple bioclimatic variables also creates the conditions to take frequencies of climate extremes into account. When the relative CEE representing the percentage of cases for each individual model run that a climate envelope was exceeded, providing a better overview of yearly climatic extremes than averaging yearly data into longer time frames.

Many of the bioclimatic variables that we found to be significant in determining species distribution correspond to those found in past studies. Dyderski et al.^47^ found that BIO5 (Precipitation seasonality), BIO7 (Temperature annual range), BIO10 (Mean temperature of the warmest quarter) and BIO18 (precipitation of the warmest month) were of the highest importance for the projected range of tree species in Europe. Walentowski et al.^59^ found BIO1 (Annual mean temperature), BIO6 (Min. temperature of the coldest month), BIO10 (Mean temperature of the warmest quarter), BIO12 (Annual precipitation), BIO18 (Precipitation of the warmest quarter) to be significant for the prediction of the occurrence of tree species in Southern Germany. While Bell & Lauenroth^60^ found Mean annual temperature (BIO1) as well as Annual precipitation (BIO12) and climatic factors similar to BIO18 and BIO19 (mean summer and mean winter precipitation) to be significant for younger life stages of trees in the United States.

Climate models consistently project a warming trend for the representative area used located within the Hamburg metropolitan area. Trends in precipitation projections are less clear. For the annual average, there are models projecting a decrease, an increase, and no considerable change in precipitation (see supplementary Fig. S11). Despite this uncertainty in annual precipitation, the CMIP6 models agree on an increase in the seasonality with a robust decrease in summer precipitation and a robust increase in winter precipitation (compare supplementary Figs. S12, S13, S14).

The upper and lower limits of the climate envelopes obtained in this study largely agree with known tolerances in other bodies of work. Values for Beech found by various studies^61–63^largely show comparably temperature and precipitation ranges for Beech. Although Kapeller et al.^64^ finds much a much lower cut-off point for mean annual precipitation for spruce, around 550 mm. For oak, studies show that they are more sensitive to precipitation than temperature^65,66^.

When looking at the general trend in CEEs for all tree species, we could observe a difference between the two variables for Annual Temperature and Precipitation(BIO1, BIO12) and the other significant variables for Temperature seasonality, Maximum temperature of the warmest month, Precipitation seasonality and Precipitation of the warmest and coldest quarters (BIO4, BIO5 , BIO15, BIO18 and BIO19). The former showing lower frequency of CEE than the latter. The latter variables all quantify some form of seasonal differences and show a greater effect of the warmer and drier conditions predicted by the ESMs. The larger increase in the seasonally distinct variables as opposed to the yearly averages shows an increase in extreme events, these have a disproportionately large impact on forest mortality when compared to increases in the average^33,67^.

These results can be compared to similar studies on the mortality risks of tree species in Europe under a changing climate. Paul et al.^68^ finds that survival probabilities for spruce as compared to beech are that spruce has a lower survivability in future climate scenarios, but also shows that there is not a large difference between survival probabilities of beech and those of spruce.

Using climate envelopes, allows us to assess the suitability of climate conditions for tree species on a broad scale, but this is a limited approach that does not include several critical factors that also have a great influence on future tree mortality. These factors, such as soil water capacity^69,70^, inter-species competition^71^, frost onset and duration^72^, diseases outbreaks and insect plagues^19–24^ must also be considered in order to perform a more complete analysis of tree species vulnerability under future climate change. It is important to asses the attribution of both climate and the previously mentioned environmental factors on future tree vulnerability, possibly through an attribution analysis, as is often done for climate and extreme events^73–75^.

Within our approach, we must also note that, the range of the historical data (1960–2020) itself includes a significant time period with increasing warming, As a result, trees that have germinated well in the past, did so in a climate significantly different from today. However, we found that shortening the reanalysis data to the time period 1960–1990 did not result in a significant change in the results. Another important note is that future climate projections were obtained for a one area in the Hamburg metropolitan region, the scope of this study was limited to asses the viability of this method for comparing species vulnerability without the need to filter out location specific compounding variables. In the future, a more comprehensive assessment is needed covering a larger area in order to be able to generalize these results. it is also necessary to note that many steps such as the national forest inventories, tree species distribution model and the future climate data contain significant uncertainties. For each step these uncertainties must be quantified and analysed.

It is also important to note, that this approach makes no differentiation whether individuals have been planted or occur naturally in a certain area. Many species, especially spruce and pine are used in European commercial forests on a large scale, and may be located well beyond their potential natural range.

## Conclusion

The results presented in this research, among of all the tree species included in this study, spruce is especially vulnerable to changes in yearly average precipitation, as well as the seasonal distribution of temperature and precipitation. When using a climate envelope approach, it became obvious that spruce has a far lower tolerance for dry and warm conditions than the other tree species included in this study. If we extrapolate current emission reduction policies, which suggest a warming of about 3.0 $^\circ$C until the end of the century^76^, we can show that not only Spruce but also other tree species will experience frequent potential climate stresses. Our study suggests that in such a climate current forest species composition can not be maintained. Current mitigation scenarios that would reach the 1.5 $^\circ$C limit of the Paris Agreement rely heavily on nature based solutions to reduce atmospheric CO_2_ concentrations. But even when lower warming rates are realized, forests will experience considerable stress, which will have an affect their carbon sink potential.

Our study highlights that, the warmer and drier summers, a trend also seen by more extensive climate projections throughout a large part of Europe^77,78^, will, within a few decades, surpass the tolerance levels of many important commercial tree species in central Europe. This climatic trend is increasing faster than shown in yearly average values, which necessitates a move away from yearly averages and towards seasonal, or even shorter temporal range climate values in order to reliably estimate tree species climate vulnerability. These more accurate assessments will aid in future adaptation and risk minimization measures as increase in the vulnerability of tree species will have severe ecological^79–81^, social^82^ and economic^8,83,84^ consequences, and will negatively affect forest carbon sequestration potential. We therefore also suggest that all available knowledge and methodology should be considered to both manage and minimize risk, such as fire suppression^85,86^, wind throw prevention^87^ and insect plague management^88,89^ as well as climate adaptation methods^90,91^ in order to maintain healthy and productive forest ecosystems in the future. However, there is not a one-for-all solution for forest adaptation as forests growth and mortality often often strongly depend on local conditions^92,93^.

### Supplementary Information

Supplementary Information 1.

Supplementary Information 2.

Supplementary Information 3.

Supplementary Information 4.

Supplementary Information 5.

Supplementary Information 6.

Supplementary Information 7.

Supplementary Table 1.

## Data Availability

Data obtained from the analysis of tree species occurrence, historical climate data as well as future climate scenarios and data of the calculated climate envelopes can be accessed at https://doi.org/10.25592/uhhfdm.13722.
