# Supplementary figures and images for: Using climate envelopes and earth system model simulations for assessing climate change induced forest vulnerability
Source: Sci Rep. 2024 Jul 24;14:17076. doi: 10.1038/s41598-024-68181-5 (PMC11269643; doi:10.1038/s41598-024-68181-5)

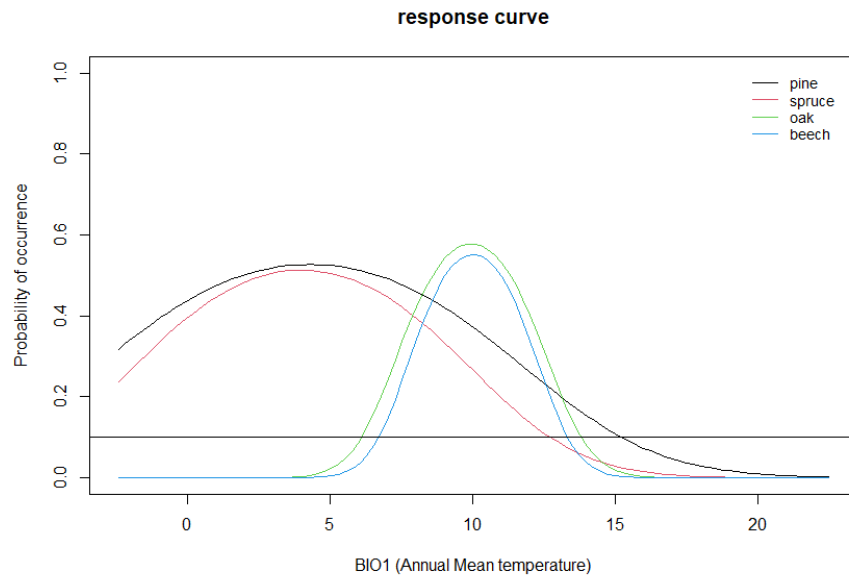

**Fig. 1:** SDM response curve for BIO1

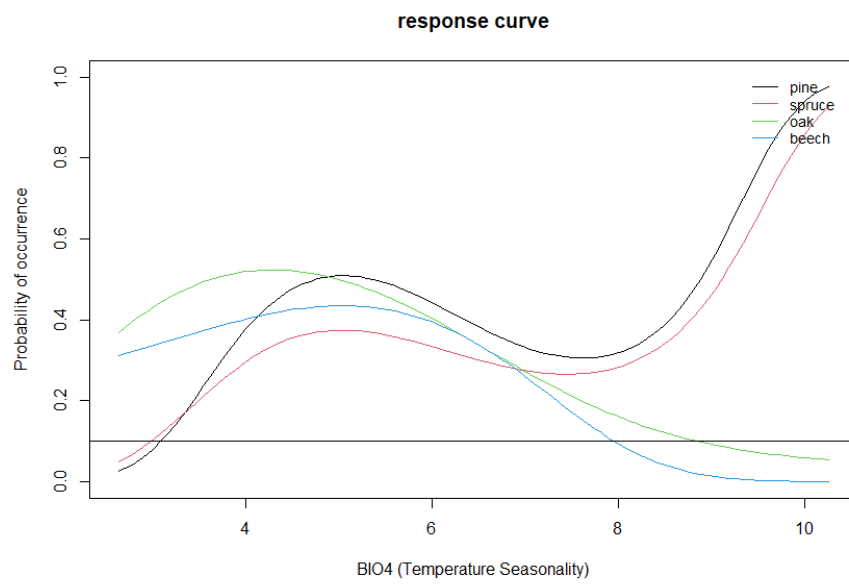

**Fig. 2:** SDM response curve for BIO4

Supplement: Supplementary file 1 — Supplementary Information 1. [file 41598_2024_68181_MOESM1_ESM.pdf]

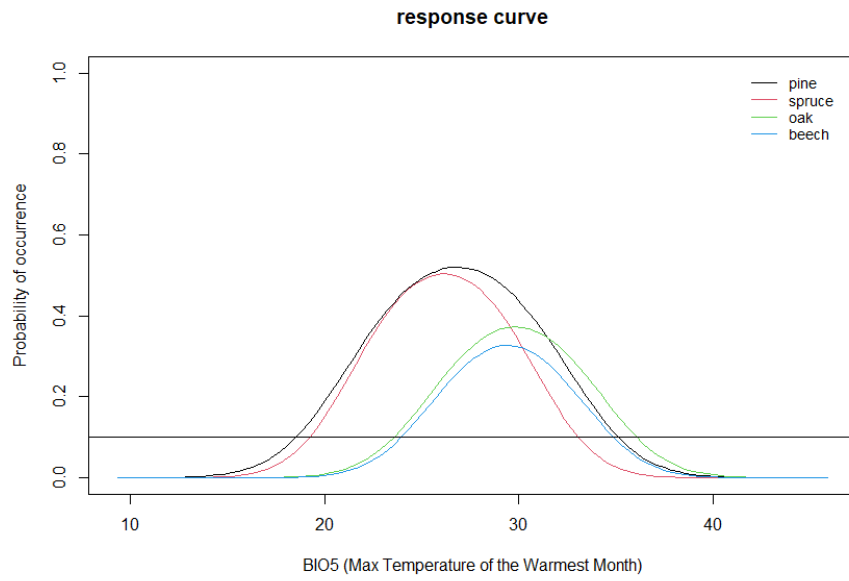

**Fig. 3:** SDM response curve for BIO5

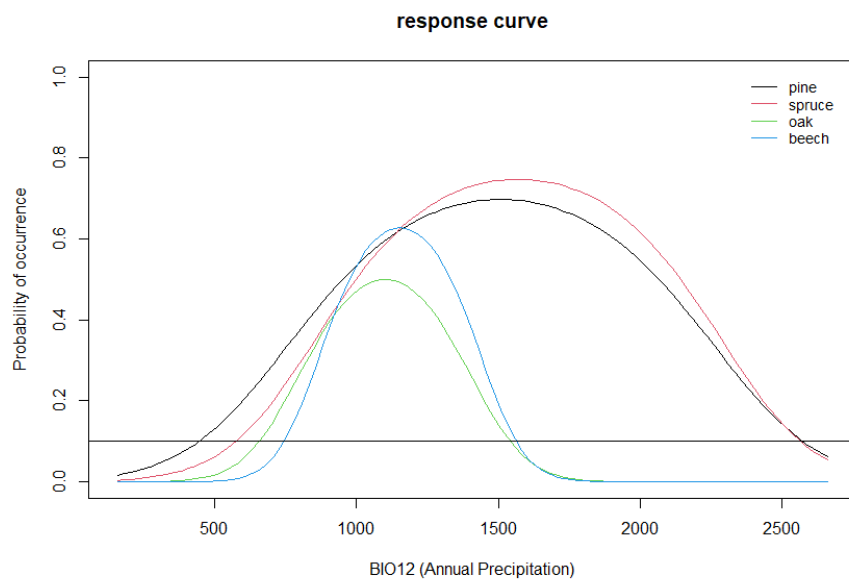

**Fig. 4:** SDM response curve for BIO12

Supplement: Supplementary file 2 — Supplementary Information 2. [file 41598_2024_68181_MOESM2_ESM.pdf]

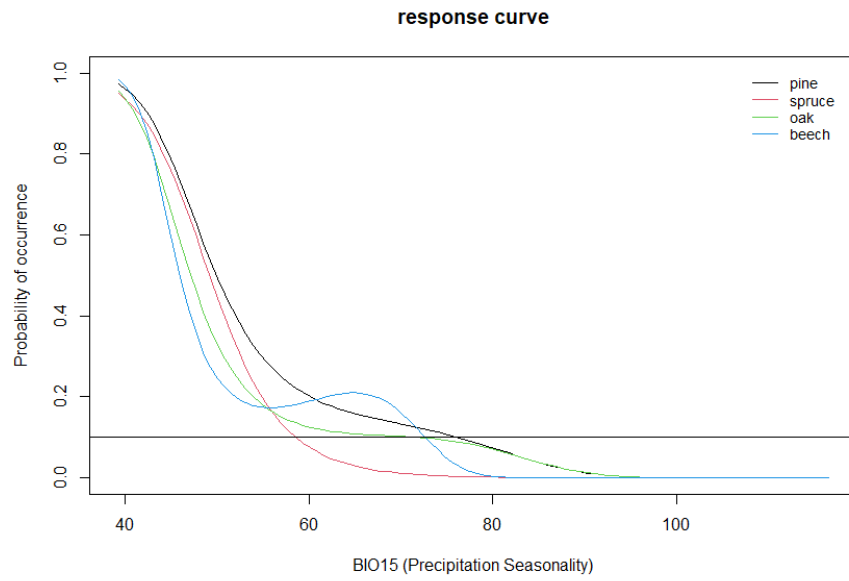

**Fig. 5:** SDM response curve for BIO15

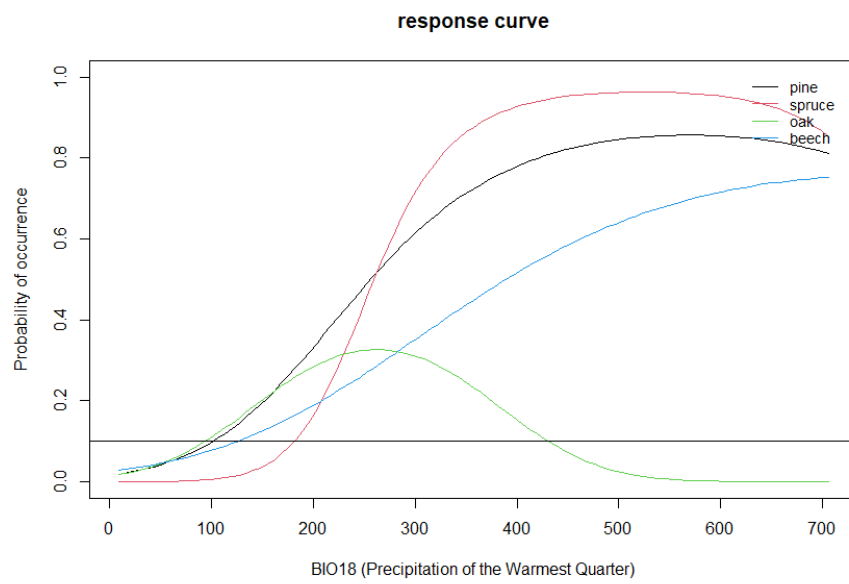

**Fig. 6:** SDM response curve for BIO18

Supplement: Supplementary file 3 — Supplementary Information 3. [file 41598_2024_68181_MOESM3_ESM.pdf]
