# Supplementary material for: Using climate envelopes and earth system model simulations for assessing climate change induced forest vulnerability
Source: Sci Rep. 2024 Jul 24;14:17076. doi: 10.1038/s41598-024-68181-5 (PMC11269643; doi:10.1038/s41598-024-68181-5)
Supplement: Supplementary file 5 — Supplementary Information 5. [file 41598_2024_68181_MOESM5_ESM.pdf]

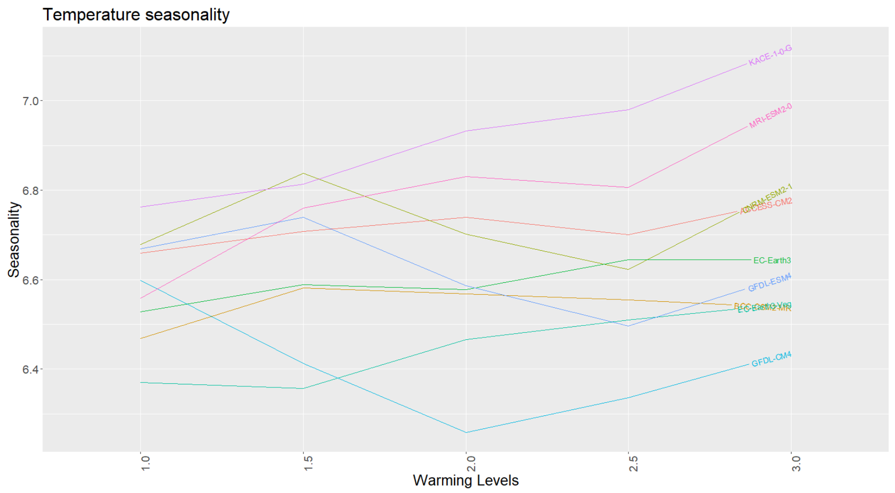

Fig. S9: Average model results per model of all the selected models for BIO4

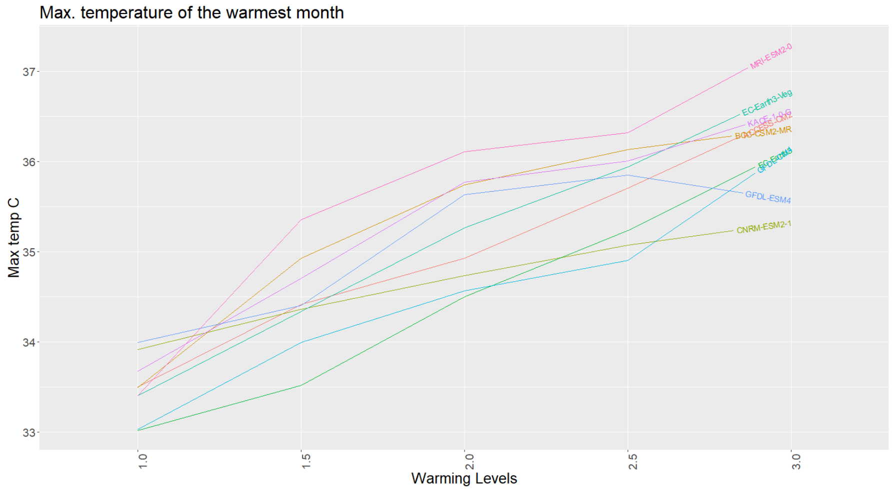

Fig. S10: Average model results per model of all the selected models for BIO5
