# Supplementary material for: Using climate envelopes and earth system model simulations for assessing climate change induced forest vulnerability
Source: Sci Rep. 2024 Jul 24;14:17076. doi: 10.1038/s41598-024-68181-5 (PMC11269643; doi:10.1038/s41598-024-68181-5)
Supplement: Supplementary file 7 — Supplementary Information 7. [file 41598_2024_68181_MOESM7_ESM.pdf]

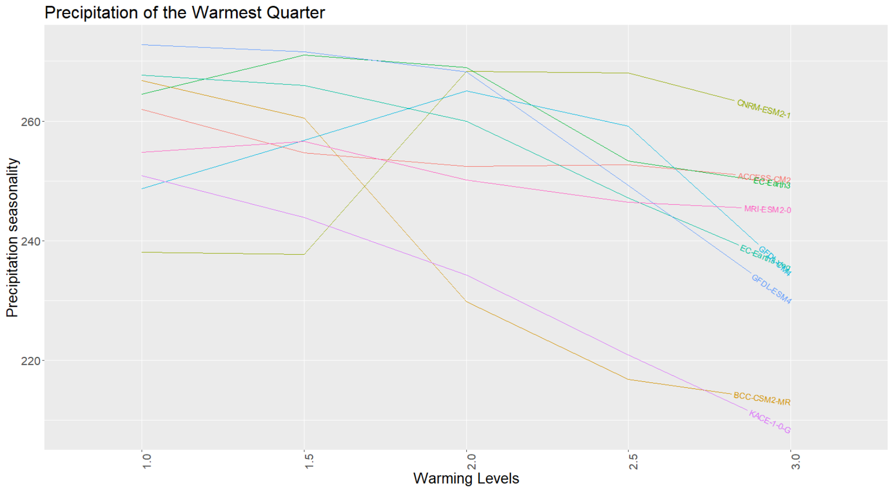

Fig. S13: Average model results per model of all the selected models for BIO18

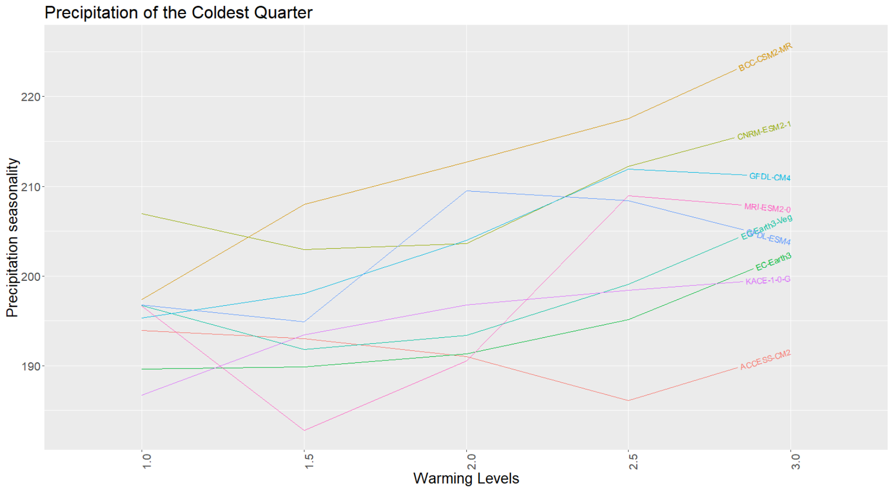

Fig. S14: Average model results per model of all the selected models for BIO19
