# Supplementary material for: Using climate envelopes and earth system model simulations for assessing climate change induced forest vulnerability
Source: Sci Rep. 2024 Jul 24;14:17076. doi: 10.1038/s41598-024-68181-5 (PMC11269643; doi:10.1038/s41598-024-68181-5)
Supplement: Supplementary file 8 — Supplementary Table 1. [file 41598_2024_68181_MOESM8_ESM.pdf]

**Table S1**: NMDS1 output table with the 1$^{st}$ and 2$^{nd}$ axis results (NDSM1 and NDSM2) with accompanying $r^2$ and P values

| | NMDS1 | NMDS2 | $r^2$ | Pr(>r) |
|---|---|---|---|---|
| BIO1 | 0.99922 | -0.03951 | 0.5037 | 0.001 |
| BIO2 | -0.63429 | 0.77309 | 0.0402 | 0.001 |
| BIO3 | 0.9604 | -0.27863 | 0.2864 | 0.001 |
| BIO4 | -0.86684 | 0.49859 | 0.203 | 0.001 |
| BIO5 | 0.92874 | 0.37074 | 0.291 | 0.001 |
| BIO6 | 0.97283 | -0.23153 | 0.3727 | 0.001 |
| BIO7 | -0.84319 | 0.53762 | 0.1686 | 0.001 |
| BIO8 | 0.94198 | 0.33566 | 0.1281 | 0.001 |
| BIO9 | 0.99967 | -0.02566 | 0.3982 | 0.001 |
| BIO10 | 0.97678 | 0.97678 | 0.39 | 0.001 |
| BIO11 | 0.98773 | -0.15615 | 0.4379 | 0.001 |
| BIO12 | 0.02334 | -0.99973 | 0.0694 | 0.001 |
| BIO13 | 0.08588 | -0.99631 | 0.0302 | 0.001 |
| BIO14 | -0.3059 | -0.95206 | 0.1138 | 0.001 |
| BIO15 | 0.35868 | 0.93346 | 0.062 | 0.001 |
| BIO16 | -0.03197 | -0.99949 | 0.0436 | 0.001 |
| BIO17 | -0.02652 | -0.99965 | 0.0977 | 0.001 |
| BIO18 | -0.52642 | -0.85023 | 0.1682 | 0.001 |
| BIO19 | 0.63275 | -0.77436 | 0.0502 | 0.001 |
